# Preliminary analysis of the diagnostic value and mechanism of action of TGFbI and S100A4 in hepatocellular carcinoma

**DOI:** 10.5937/jomb0-54550

**Published:** 2025-06-13

**Authors:** Liang Zhao, Qinghua Shu, Bowen Sha, Miao Wu, Yufeng Zhang

**Affiliations:** 1 Nanjing University of Chinese Medicine, The Second Hospital of Nanjing, Department of General Surgery, Nanjing, China

**Keywords:** TGFbI, S100A4, hepatocellular carcinoma, ferroptosis, diagnostics, TGF I, S100A4, hepatocelularni karcinom, feroptoza, dijagnostika

## Abstract

**Background:**

The aim was to analyse the diagnostic value of transforming growth factor-beta-induced protein (TGF I) and S100 calcium-binding protein A4 (S100A4) on hepatocellular carcinoma (HCC) and to explore further the effects of TGF I and S100A4 on ferroptosis in HCC cells.

**Methods:**

We retrospectively analysed 76 patients with HCC admitted to our hospital from October 2022 to June 2023 and detected the differences in the expression of TGF I and S100A4 in cancerous tissues and paracancerous tissues to analyse their diagnostic and prognostic assessment value for HCC. Additionally, the HCC cell line HepG2 was purchased and transfected with TGF I and S100A4 abnormal expression plasmids to check changes in cell viability, oxidative stress damage, mitochondrial damage, and ferroptosis.

**Results:**

TGF I and S100A4 were upregulated in HCC tissues (P<0.05), and their combined detection exhibited excellent diagnostic effects for HCC. The levels of TGF I and S100A4 in patients who died prognostically were higher than those in surviving patients (P<0.05). An increase in the levels of TGF I and S100A4 indicates an elevated risk of prognostic death in patients. Upregulating TGF I and S100A4 expression in cell experiments activated HepG2 activity, inhibited apoptosis, mitochondrial and oxidative stress damage, and improved cell ferroptosis.

**Conclusions:**

TGF I and S100A4 are elevated in HCC and can potentially be clinical diagnostic indicators of HCC.

## Abstract (sr)

**Uvod:**

Cilj je bio da se analizira dijagnostička vrednost proteina izazvanog transformišućim faktorom rasta beta (TGF I) i proteina koji veže kalcijum S100A4 (S100A4) kod hepatocelularnog karcinoma (HCC) i da se dalje istraže efekti TGF I i S100A4 na feroptozu u ćelijama HCC.

**Metode:**

Retrospektivno smo analizirali 76 pacijenata sa HCC koji su lečeni u našoj bolnici od oktobra 2022. do juna 2023. godine i utvrdili razlike u ekspresiji TGF I i S100A4 u tumorskom i paratumorskom tkivu kako bismo procenili njihovu dijagnostičku i prognostičku vrednost za HCC. Pored toga, kupljena je ćelijska linija HCC HepG2, koja je transficirana plazmidima sa abnormalnom ekspresijom TGF I i S100A4, kako bi se ispitale promene u ćelijskoj održivosti, oštećenja od oksidativnog stresa, mitohondrijalna oštećenja i feroptoza.

**Rezultati:**

TGF I i S100A4 su bili povećani u HCC tkivima (P<0,05), a njihova kombinovana detekcija pokazala je izvanredne dijagnostičke efekte za HCC. Nivoi TGFI i S100A4 kod pacijenata koji su preminuli bili su viši u odnosu na pacijente koji su preživeli (P<0,05). Povećanje nivoa TGF I i S100A4 ukazuje na veći rizik od smrtne prognoze kod pacijenata. Povećana ekspresija TGF I i S100A4 u eksperimentima na ćelijama aktivirala je HepG2 ćelije, inhibirala apoptozu, mitohondrijalno i oksidativno oštećenje i poboljšala ćelijsku feroptozu.

**Zaključak:**

TGF I i S100A4 su povišeni u HCC i mogu da budu potencijalni kliničko-dijagnostički pokazatelji za HCC.

## Introduction

Hepatocellular carcinoma (HCC) averages more than 780,000 new cases per year, of which the 5-year mortality rate for patients with advanced HCC can reach 60% to 70% [1]. However, with the advancement of modern medical technology, various HCC treatment modalities such as surgical resection, liver transplantation, and liver-targeted therapy have been continuously refined to notably prolong patient prognostic survival [2], achieving more secure, effective, and stable diagnosis and treatment of HCC remains a key priority that requires continuous efforts from clinical researchers [3]. Ferroptosis is a newly discovered form of iron-mediated regulated cell death distinct from apoptosis, necrosis, and autophagy. It is primarily associated with iron-dependent lipid peroxidation metabolism. It participates in the development of cellular oxidative damage and redox imbalance, holding significant importance in tumorigenesis, progression, and multidrug resistance [4]. Currently, research has confirmed that ferroptosis upregulated factors (FUF) and ferroptosis down-regulated factors (FDF) in HCC regulate ferroptosis by influencing glutathione (GSH) synthesis [5]. Consequently, the study of ferroptosis in diagnosing and treating HCC has drawn extensive attention from researchers.

In this regard, we tried to identify new specific molecular markers based on ferroptosis in HCC. In asingle-cell transcriptome analysis of LC by Ding Z et al. [6] transforming growth factor (TGF)-betainduced protein (TGFβI) and S100 calcium-binding protein A4 (S100A4), associated with macrophage polarisation, have attracted our attention. TGFβI is a secreted protein induced by TGF-β and plays a vital role in cell biological behaviours such as cell proliferation, angiogenesis, and apoptosis [7]. Recently, the research by Huang H [8] indicates that TGFβI exhibits abundant immunosuppressive effects in the tumour immune microenvironment and can potentially become a new cancer treatment target. In addition, in an animal experiment on ovariectomised rats with echinochrome A, Kim JM [9] also found that an increase in TGFβI level is accompanied by the inhibition of ferroptosis. These studies have laid a foundation for applying TGFβI in diagnosing and treating HCC. However, as TGFβI has been demonstrated to be abnormally expressed in multiple tumour diseases [10] [11], it is still challenging to achieve an accurate early diagnosis of HCC solely relying on TGFβI. S100A4, as a communication molecule closely related to TGFβI, is considered an essential energy source in TGFβI-induced fibrosis [12]. In chronic ethanolinduced fatty liver, S100A4 has been confirmed to be essential in regulating inflammatory responses and lipid accumulation [13]. Although there is no study confirming the relationship between TGFβI and HCC, recently in a study by Sun H et al. [14], they also mentioned that S100A4 can activate HCC metastasis, which lays the foundation for the future clinical application of TGFβI and S100A4 in HCC.

We believe that combined diagnosis using TGFβI and S100A4 can achieve more accurate early assessment of HCC, but currently, there is still a lack of relevant research to support our view. In this study, we aim to verify the future diagnostic and therapeutic applications of TGFβI and S100A4 in HCC by analysing the evaluation effect of TGFβI plus S100A4 on the progression of HCC and their influence on ferroptosis in HCC, thereby providing references for clinical practice and subsequent research.

## Materials and methods

### Research subjects

The sample size required for the study was calculated according to sample size (N) = Z^2^ × [P × (1-P)]/E^2^. Parameters were set: statistic (Z) = 1.64, probability (P) = 0.5, error (E) = 10%, and the result of the calculation N = 67. 76 HCC patients admitted to our hospital from October 2022 to June 2023 were selected as the research subjects for retrospective analysis, 42 males and 34 females, mean age (68.68±4.78) years. Inclusion criteria: Patients over 18 years old, with a definite diagnosis of HCC by pathological biopsy, primary tumours, surgical treatment in our hospital after admission, complete baseline data, post-discharge follow-up data and TNM stage I–III were included. Exclusion criteria: Patients with a previous history of other malignant tumours, those who died within one month after surgery, those who received neoadjuvant chemotherapy, targeted therapy, or other intervention treatments before surgery, or those with combined dysfunction of important organs such as the heart, lungs, and kidneys, were excluded. The study involving human subjects complied with the Declaration of Helsinki. It was approved by the ethical committee of the SecondHospital of Nanjing, Nanjing University of Chinese Medicine (No.2 022-LY-js004), and all participants provided written informed consent.

### Sampling and testing

Part of the HCC tissue and the corresponding adjacent tissue (at a distance greater than 5 cm from the tumour) obtained intraoperatively were fixed in 10% neutral formaldehyde for 12 hours, paraffinembedded, sectioned, and baked at 60 °C for 3 hours. Immunohistochemical (IHC) staining of the sections was performed after dewaxing and hydration, followed by antigen retrieval in citrate repair solution. 20 minutes of blocking with hydrogen peroxide and 2 hours of sealing with 3% goat serum at room temperature. TGFβI and S100A4 primary antibodies (1:500) (Abcam) were then added dropwise to incubate at 4 °C for 16 hours. The positive expressions of TGFβI and S100A4 were observed under the microscope after colour development and mounting (the scoring criteria are shown in Table 1; positive expression = staining intensity score × positive cell distribution score).

**Table 1:** Criteria for evaluation of IHC staining results.

Score	0	1	2	3	4
Intensity of<br>staining	None	Light<br>yellow	Brownish<br>yellow	Brownish<br>brown	–
Distribution<br>of positive<br>cells	<5%	6–25%	26–50%	51–75%	>76%

### Quantitative Real-Time Polymerase Chain Reaction (qRT-PCR)

20 mg of tissue sample was collected to add with a lysis solution, grind thoroughly with a grinder, and centrifuge (3000 rpm/min, 5 min) for supernatant isolation. The total RNA of the tissue was extracted from tissues with a Trizol kit (Thermo Fisher) and reverse transcribed to synthesise cDNA for a PCR reaction with a SYBR GREEN Master MIX kit (Thermo Fisher). Reaction conditions: 95 °C for 5 minutes, 94°C for 30 seconds, 62°C for 25 seconds, 72°C for 30 seconds, for a total of 40 cycles. The primer sequences were designed by TinyGene Bio-Tech (Shanghai) Co., Ltd. (Table 2), and TGFβI and S100A4 mRNA levels were calculated with GAPDH as an internal reference.

**Table 2:** Primer sequences for PCR.

	Forward primer (5’-3’)	Reverse primer (5’-3’)
TGFβI	GACGTTCGCCATAACCAAGT	CTGCAGGTTCTCAATGCAAA
S100A4	CCCTGGATGTGATGGTGT	GTTGTCCCTGTTGCTGTC
GAPDH	ATGTTCGTCATGGGTGTGAAC	ATGGACTGTGGTCATGAGTCC

### Prognostic follow-up

All patients underwent a one-year prognostic follow-up. This follow-up was implemented through regular re-examinations, with patients required to have an interval of no more than two months between re-examinations. The follow-up content included the patients’ survival status.

### Cell materials

HepG2, a human LC cell line, was purchased from Guangzhou Jennio Biotech Co., Ltd. and cultured in a supporting medium. Passage was performed every 2 to 3 days, and the fifth-generation cells were selected for subsequent experiments.

### Cell transfection

HepG2 cells in good growth condition were seeded in 6-well plates at a density of 3×10^5^ cells per well and randomly divided into six groups: TGFβI empty vector control group (TGFβI-NC), TGFβI overexpression group (TGFβI-OE), TGFβI small interfering RNA group (TGFβI-si-RNA), S100A4 empty vector control group (S100A4-NC), S100A4 overexpression group (S100A4-OE), and S100A4 small interfering RNA group (S100A4-si-RNA). The Guangzhou Paizhen Biotechnology Co. was entrusted to design and construct TGFβI and S100A4 interference expression plasmids. Subsequently, the overexpression plasmids and siRNA sequences were transfected into the corresponding groups of cells following the Lipo8000 transfection kit (Thermo Fisher) recommendations.

### Transfection effect verification

The transfection efficacy was verified by detecting post-transfection TGFβI and S100A4 protein expression using Western blot. Cells were lysed on ice for 20 minutes with RIPA lysis buffer (Thermo Fisher), and the protein concentration was determined via BCA assay (Merck). Following SDS-PAGE (Thermo Fisher) separation, the proteins were transferred onto a PVDF (Sigma-Aldrich) membrane. A 5% skim milk solution was then employed for blocking at room temperature for 2 hours. Subsequently, the corresponding primary antibodies TGFβI (1:1000), S100A4 (1:1000), and GAPDH (1:2000) (Abcam) were added to incubate overnight at 4°C. Following membrane washing with TBST thrice, a horseradish peroxidase-labelled IgG secondary antibody (1:5000) (Abcam) was added and incubated for 1 hour. Enhanced chemiluminescence (ECL) luminescence liquid (MCE) was utilised for development. With GAPDH as an internal reference, ImageJ software was employed for band analysis. Additionally, GPX4 (1:1000), p53 (1:1000), and ACSL4 (1:1000) protein levels were detected to assess cellular ferroptosis.

### MTT assay for cell proliferation

Cells were seeded into 96-well plates at 7×10^3^/well and cultured overnight. At 24, 48, 72, and 96 hours, 100 μL of 0.1% 3-(4,5)-dimethylthiahiazo(- z-y1)-3,5-di- phenytetrazoliumromide (MTT) (Beyotime Biotechnology) solution was added to each well. After incubation for 4 hours, the absorbance value at optical density (OD)=490 nm was measured, and a cell growth curve was plotted.

### Flow cytometry for apoptosis detection

Cells from each group were digested and collected, resuspended, and adjusted to a concentration of 1×10^6^/mL. After PBS cleaning, the supernatant was discarded by centrifugation (800 rpm/min), and 5 mL each of Annexin V-Fluorescein isothiocyanate (FITC) and propidium iodide (PI) reagent (Sigma-Aldrich) were added. After incubation at room temperature for 15 minutes, the apoptosis rate and cycle changes were detected by flow cytometry (CytPix, Thermo Fisher).

### Oxidative stress assay

0.2 mL of malondialdehyde (MDA) working solution was added into the cells, followed by heating in a boiling water bath for 15 minutes and supernatant collection via centrifugation. The OD value at 532 nm was measured with a microplate reader, representing the MDA content. Subsequently, cells were collected and added to a protein removal reagent three times the volume of the cells. Repeated freezethaw cycles were performed twice using liquid nitrogen and a 37°C water bath. The supernatant was obtained by centrifugation again. Finally, the OD value at 412 nm was measured at room temperature, representing the GSH level.

### Mitochondrial membrane potential experiment

1 mL of cell culture medium and 1 mL of preprepared JC-1 staining working solution (Abcam) was added to the cells, followed by 20 minutes of cell incubator incubation at 37°C. After incubation, the supernatant was aspirated, and the cells were washed twice with the prepared JC-1 staining buffer (1×). Carbonyl cyanide m chlorophenylhydrazone (CCCP) (Abcam) was a positive control for decreasing mitochondrial membrane potential. Next, CCCP from the kit was added to the cell culture medium at a ratio of 1:1000 and diluted to 10 μmol/L to treat the cells for 20 minutes. Finally, observation was conducted under a fluorescence microscope, and the images’ red/ green fluorescence intensity ratio was analysed using Image J software (National Institutes of Health).

### Reactive oxygen species (ROS) and Fe2+ detection

2’,7’-Dichlorodihydrofluorescein diacetate (DCFHDA) (Sigma-Aldrich) and SiRhoNox-1 (Sigma-Aldrich) were used to evaluate intracellular ROS and Fe2+ levels. DCFH-DA and SiRhoNox-1 were diluted with serum-free culture solution at a ratio of 1:1000 to achieve a final concentration of 10 mmol/L, which were then used to incubate cells in each group at 37°C for 20 minutes. ROS and Fe2+ fluorescence intensities were observed using a fluorescence microscope. Fluorescence intensity = sum of fluorescence intensity in the area (IntDen)/Area.

### Statistical methods

All experiments in this research were repeated three times. The results were statistically analysed using SPSS 24.0 software (IBM). The Shapiro-Wilk test was utilised to examine the normal distribution of the data. For normally distributed data (mean ± standard deviation), repeated measures analysis of variance and Bonferroni’s within-group test were employed for comparison. The Kruskal-Wallis H test compared non-normally distributed data [median (interquartile range)]. Diagnostic value was analysed using receiver operating characteristic (ROC) curves. Survival rates were calculated using the Kaplan-Meier method, and the comparison of survival rates was performed using the Log-rank test. A P-value less than 0.05 was considered statistically significant.

## Results

### TGFβI and S100A4 are highly expressed in HCC

**Figure 1:**
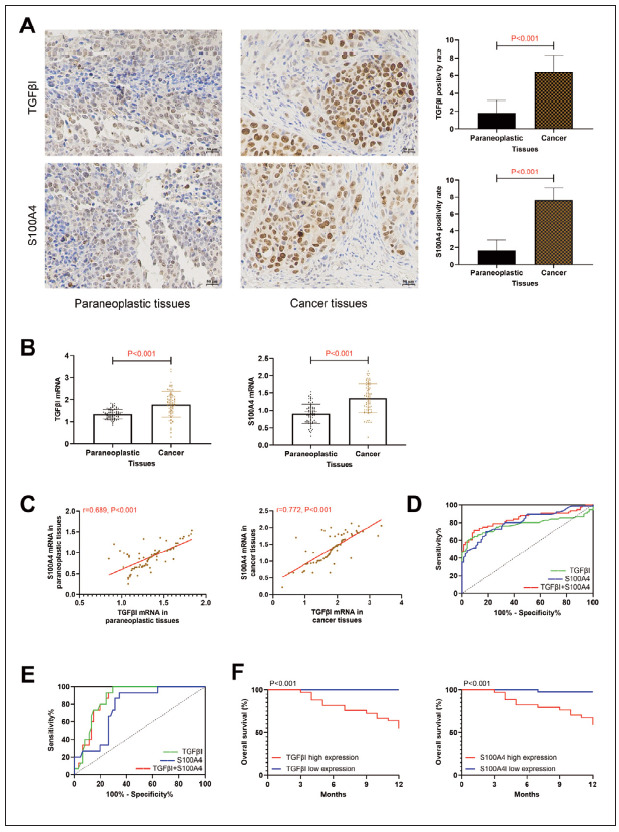
Clinical significance of TGFβI and S100A4.

Subsequently, the expression of TGFβI and S100A4 was detected. Higher positive rates of TGFβI and S100A4 were detected in HCC tissues than in adjacent tissues (P<0.05), suggesting abnormally upregulated TGFβI and S100A4 in HCC (Figure 1A). Similarly, PCR detection showed that TGFβI and S100A4 mRNA levels in cancer tissues were (1.79±0.58) and (1.35±0.42) respectively, which were higher compared to adjacent tissues (P<0.05, Figure 1B). Meanwhile, TGFβI and S100A4 mRNA expression showed a positive correlation trend in both cancer and adjacent tissues (P<0.05, Figure 1C). According to ROC curve analysis, when Log(P) of the combined detection [Log(P)=6.350+(-1.884× TGFβI)+(-3.185×S100A4)] of TGFβI and S100A4 was <0.43, the sensitivity and specificity for diagnosing the occurrence of HCC were 71.05% and 90.79%, respectively (P<0.05, Figure 1D, Table 3). Prognostic follow-up successfully tracked all research subjects, among which 15 patients died. The comparison revealed that TGFβI and S100A4 mRNA levels were higher in patients who died prognostically than in surviving patients (P<0.05, Figure 1B). ROC curve analysis indicated that the combined detection [Log(P)=6.935+(-2.320×TGFβI)+(-0.628×S100A4)] of TGFβI and S100A4 also exhibited excellent evaluation effects on prognostic survival (Figure 1E, Table 3). Based on the cut-off value, patients were categorised into TGFβI high (TGFβI mRNA>1.88, n=33). Low expression group (TGFβI mRNA 1.88, n=43) and S100A4 high (S100A4 mRNA>1.41, n=34) and low expression group (S100A4 mRNA 1.41, n=42), respectively, and drew prognostic survival curves. The high-expression groups of TGFβI and S100A4 were found to have lower prognostic survival rates than the low-expression groups (P<0.05, Figure 1F).

**Table 3:** Effectiveness of TGFβI and S100A4 in the assessment of HCC. Note: Area under curve (AUC), 95% confidence interval (95%CI).

Projects	Indicators	AUC	95%CI	Std. Error	P	Cut-off	Sensitivity (%)	Specificity (%)
Diagnosing the<br>occurrence of<br>HCC	TGFβI	0.777	0.695–0.858	0.041	<0.001	>1.75	59.21	96.05
	S100A4	0.810	0.741–0.878	0.035	<0.001	>1.11	69.74	81.58
	TGFβI+S100A4	0.842	0.776–0.908	0.034	<0.001	<0.43	71.05	90.79
Diagnosis of<br>prognostic<br>death in HCC	TGFβI	0.871	0.792–0.949	0.040	<0.001	>1.88	100.0	70.49
	S100A4	0.769	0.656–0.883	0.058	<0.001	>1.41	93.33	65.57
	TGFβI+S100A4	0.865	0.785–0.944	0.041	<0.001	<0.85	100.0	70.49

A: IHC detection of TGFβI and S100A4 expression in HCC (40×), TGFβI and S100A4 were highly expressed in cancer tissues. B: PCR detection of TGFβI and S100A4 expression in HCC, TGFβI and S100A4 were highly expressed in cancer tissues. C: Correlation of TGFβI and S100A4 in paracancerous and cancerous tissues, TGFβI and S100A4 were positively correlated. D: ROC curves of TGFβI and S100A4 combined diagnosis of HCC occurrence. E: ROC curve of prognostic death of HCC diagnosed by the combination of TGFβI and S100A4. F: Prognostic generation curves in patients with high/low expression of TGFβI, S100A4.

### Elevated expression of TGFβI and S100A4 promotes HCC activity

**Figure 2:**
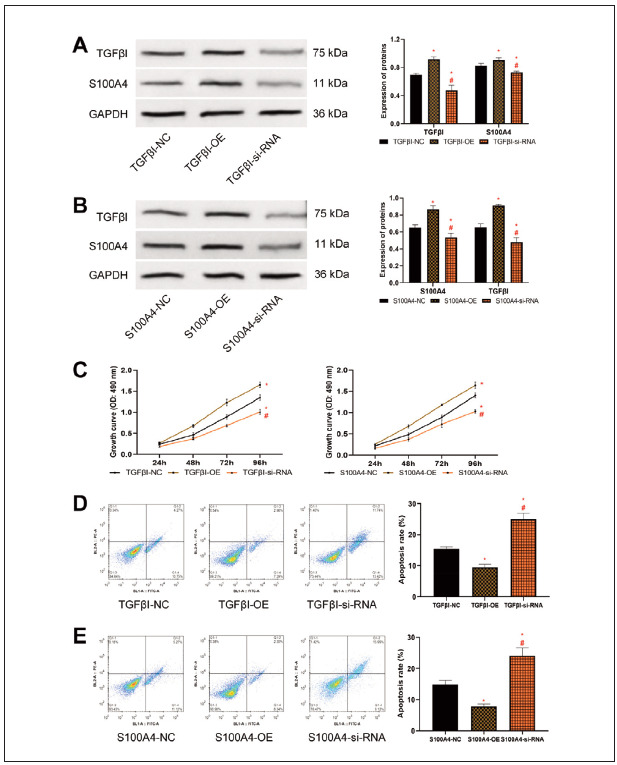
Impact of TGFβI and S100A4 on HCC cell activity. A: Interference with TGFβI and S100A4 protein expression in HepG2 after TGFβI expression B: Expression of TGFβI and S100A4 proteins in HepG2 after interference with S100A4 expression. C: Growth curve of HepG2 after interference with TGFβI and S100A4 expression, elevated expression of TGFβI and S100A4 promoted cell proliferation. D and E: Apoptosis rate of HepG2 after interference with TGFβI and S100A4 expression, elevated expression of TGFβI and S100A4 inhibited apoptosis. Note: * denotes P<0.05 with the corresponding NC group, # denotes P<0.05 with the corresponding si-RNA group.

To further clarify the mechanism of action of TGFβI and S100A4 in HCC, we transfected abnormal expression plasmids of TGFβI and S100A4 to interfere with their expression. We observed changes in HCC cell biological behaviour. First, TGFβI and S100A4 protein expression after transfection was detected. Higher TGFβI protein expression in the TGFβI-OE group versus the TGFβI-NC group, as well as lower TGFβI protein levels in the TGFβI-si-RNA group versus the TGFβI-NC group were determined (P<0.05); S100A4 protein expression was also higher in the S100A4-OE group than in the S100A4-NC group, while it was reduced in the S100A4-si-RNA group (P<0.05), confirming the success of transfection. Moreover, with the intervention of TGFβI abnormal expression plasmids, we also found changes in S100A4 expression (the same is true for S100A4 abnormal expression plasmids) (Figure 2A), indicating a close relationship between TGFβI and S100A4. In terms of cell activity, the cell growth ability of the TGFβI-OE and S100A4-OE groups was significantly enhanced compared to the TGFβI-NC group/S100A4-NC group, while that of the TGFβI-si-RNA and S100A4-si-RNA groups was significantly reduced (P<0.05, Figure 2B and Figure 2C). Conversely, the apoptosis rate was lower in the TGFβI-OE group/S100A4-OE group compared with the TGFβI-NC group/S100A4- NC group, and that of the TGFβI-si-RNA group/S100A4-si-RNA group was higher versus the TGFβI-NC group/S100A4-NC group (P<0.05, Figure 2D, Figure 2E).

### Silencing of TGFβI and S100A4 expression promotes oxidative stress and mitochondrial damage in HCC

**Figure 3:**
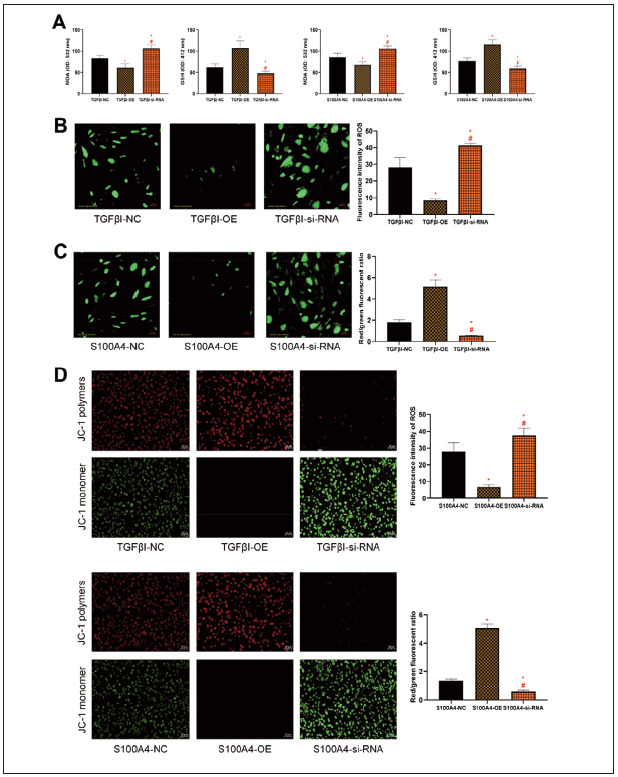
Influence of TGFβI and S100A4 on oxidative stress injury of HCC cells. A: Effects of TGFβI and S100A4 on MDA and GSH in HCC cells. B and C: Interference with ROS expression in HepG2 after TGFβI and S100A4 expression (200×). D: Mitochondrial damage in HepG2 after interference with TGFβI and S100A4 expression (200×). Note: * denotes P<0.05 with the corresponding NC group, # denotes P<0.05 with the corresponding si-RNA group.

As far as oxidative stress injury is concerned, MDA in the TGFβI-OE group/S100A4-OE group was decreased compared to the TGFβI-NC group/S100A4-NC group, while GSH in the TGFβI-si-RNA group/S100A4-si-RNA group was higher (P<0.05). MDA was higher in the TGFβI-si-RNA group/S100A4-si-RNA group than in the TGFβI-NC group/S100A4-NC group, and GSH was lower than that in the TGFβI-NC group/S100A4-NC group (P<0.05, Figure 3A). In fluorescence staining, the ROS fluorescence intensity was also lower in the TGFβI-OE group/S100A4-OE group than in the TGFβI-NC group/S100A4-NC group, while that in the TGFβI-si-RNA group/S100A4-si-RNA group was higher compared to the TGFβI-NC group/S100A4- NC group (P<0.05, Figure 3B and Figure 3C). In addition, in the detection of mitochondrial damage, we observed a higher red/green fluorescence ratio of JC-1 in the TGFβI-OE group/S100A4-OE group versus the TGFβI-NC group/S100A4-NC group, as well as a reduced JC-1 red/green fluorescence ratio in the TGFβI-si-RNA group/S100A4-si-RNA group compared to the TGFβI-NC group/S100A4-NC group (P<0.05, Figure 3D).

### Elevation of TGFβI and S100A4 expression inhibits ferroptosis in HCC

**Figure 4:**
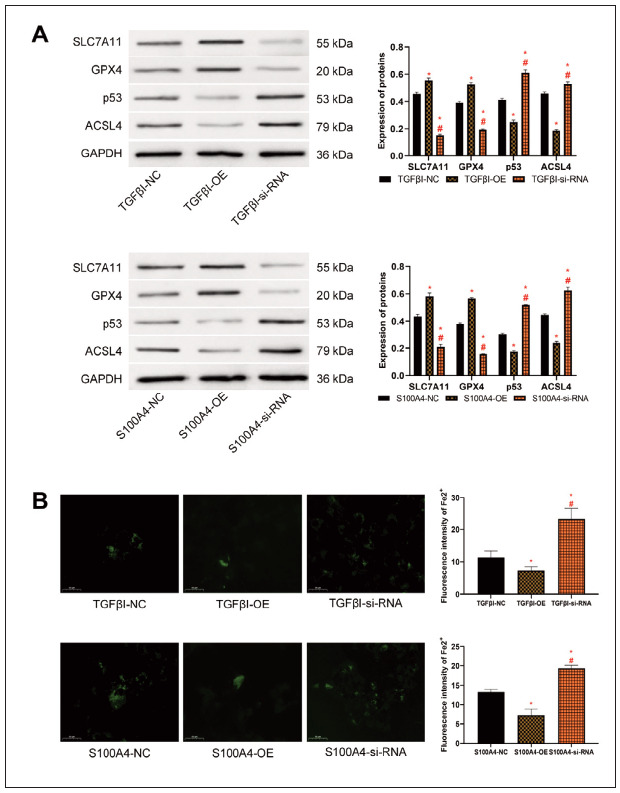
Effect of TGFβI and S100A4 on ferroptosis of HCC cells. A: Ferroptosis in HepG2 after interference with TGFβI and S100A4 expression. B: Expression of Fe2+ in HepG2 after interference with TGFβI and S100A4 expression. Note: * denotes P<0.05 with the corresponding NC group, # denotes P<0.05 with the corresponding si-RNA group.

Finally, we analysed the effects of TGFβI and S100A4 on ferroptosis in HCC cells. In comparisonwith the TGFβI-NC group/S100A4-NC group, SLC7A11 and GPX4 protein levels were increased in the TGFβI-OE group/S100A4-OE group, while p53 and ACSL4 were decreased (P<0.05). The TGFβI-si-RNA/S100A4-si-RNA groups showed lower SLC7A11 and GPX4 protein expression and higher p53 and ACSL4 protein levels than the TGFβI-NC group/S100A4-NC group (P<0.05, Figure 4A). Fluorescence staining revealed that the Fe2+ fluorescence intensity in the TGFβI-OE group/S100A4-OE group was lower than that in the TGFβI-NC groupS100A4-NC group, while that in the TGFβI-si-RNA group/S100A4-si-RNA group was higher compared to the TGFβI-NC group/S100A4-NC group (P<0.05, Figure 4B).

## Discussion

HCC still poses a serious threat to human life. Although the current multi-modal and multidisciplinary combined diagnosis and treatment, along with the formulation of individualised diagnosis and treatment plans, can enhance the 5-year survival period of HCC patients, a deeper understanding of the pathogenesis of HCC and the search for more effective diagnosis and treatment regimens are still required [15]. In this study, we found that TGFβI and S100A4 were upregulated in HCC and demonstrated excellent diagnostic effects on the development of HCC. Through cellular assays, we found that TGFβI and S100A4 could affect the activity and ferroptosis of HCC cells. These results provide new research directions for the future diagnosis and treatment of HCC.

As mentioned earlier, although the aberrant expression of TGFβI and S100A4 in LC has been confirmed in gene sequencing studies of Ding Z [6], the exact mechanism of action still needs further investigation and confirmation. Therefore, we first included clinical cases of HCC for research, trying to preliminarily confirm the exact expression of TGFβI and S100A4 in HCC. By IHC and PCR detection, it was observed that TGFβI and S100A4 were higher in cancer tissues than in adjacent tissues, confirming the abnormal upregulation of TGFβI and S100A4 in HCC, which is consistent with the gene sequencing results of Ding Z et al. [6]. In previous studies, TGFβI and S100A4 have also been found to be highly expressed in diseases such as osteoarthritis and breast cancer [16] [17]. At the same time, a positive correlation between the two is observed in both HCC cancer tissues and adjacent tissue samples, further emphasising the close connection between TGFβI and S100A4. Regarding clinical diagnosis, we found through ROC curve analysis of TGFβI and S100A4 that their combined detection exhibited a diagnostic sensitivity and specificity for HCC of 71.05% and 90.79%, respectively, with an AUC of 0.842, demonstrating excellent diagnostic efficacy, which can also provide great help for the early diagnosis of HCC in the future. Moreover, through prognostic follow-up, it was also found that TGFβI and S100A4 were closely related to the prognosis of HCC patients, and both of them showed excellent evaluation effects for prognostic death. It is suggested that in the future, the two can be used as disease assessment indicators for HCC in clinical practice to assist physicians in understanding the disease progression and prognosis of patients and formulating targeted intervention measures. Foltz JA et al. [18] proposed that excessive secretion of TGFβI and natural killer (NK) cytokines was induced after activating cytokines and tumours. The activation of TGFβI is accompanied by obvious angiogenesis and tissue fibrosis progression, which activates S100A4, mediates tissue fibrosis and recruits inflammatory responses, resulting in the same synergistic increase state [19]. However, further experimental evidence is needed to confirm the influence mechanism of the two.

In this regard, we transfected TGFβI and S100A4 abnormal expression plasmids into HCC cells and examined the alterations in their biological behaviours. First, in terms of cell activity, elevating TGFβI and S100A4 expression resulted in a significant enhancement of HCC cell activity and a reduction in apoptosis, per the results of the aforementioned clinical trials. This indicates that highly expressed TGFβI and S100A4 play the role of oncogenes in HCC. After silencing the expression of these two factors, the activity of HCC was inhibited, and apoptosis was activated. This suggests that targeted molecular therapy of silencing TGFβI and S100A4 in the future may represent a novel treatment approach for HCC. This result also corroborates the research conclusion of Huang H (8), but more studies are required to realise its clinical application.

Additionally, in numerous previous studies, the impacts of TGFβI and S100A4 on oxidative stress responses in hepatic stellate cells, macrophages, etc., have been mentioned [20] [21]. Moreover, the relationship between HCC and oxidative stress damage has been clinically recognised [22]. Therefore, we also further focused on the influence of these two factors on oxidative stress damage in HCC. Upregulating TGFβI and S100A4 expression inhibited the release of ROC in HCC cells, maintained the integrity of mitochondria, and strengthened the active state of cells. On the other hand, after silencing TGFβI and S100A4 expression, ROC in HCC cells was released in large quantities, and the mitochondrial damage was aggravated. It is hypothesised that this is also one of the mechanisms by which TGFβI and S100A4 affect cell activity. That is, the upregulation of TGFβI and S100A4 helps enhance the oxidative stress damage of HCC cells and maintain the integrity of mitochondrial function, thereby accelerating cell proliferation and promoting tumour development. Maremanda KP et al. also found a significant change in S100A4 in the process of mitochondrial fission [23], which can also preliminarily support our view.

Finally, ferroptosis, as a research hotspot in tumour cells in recent years, has been confirmed to be involved in various behaviours such as tumorigenesis, invasion, and improvement of drug resistance, and regulating ferroptosis is also considered a breakthrough in finding new tumour treatment schemes [24]. HCC is a typical tumour disease with abnormal ferroptosis, and inhibition of ferroptosis induces migration, invasion, epithelial-mesenchymal transition (EMT), and immune evasion, which are key to its progression [25]. As mentioned above, the relationship between TGFβI, S100A4, and ferroptosis has been clinically recognised. For example, calcipotriol inhibits ferroptosis of chondrocytes by blocking the TGF-b1 pathway [26], and pyruvate kinase M2 mediates S100A4 in fibroblasts to promote their proliferation [27]. However, it is not clear whether the two have any influence on ferroptosis in HCC. In this study, we found that after upregulating TGFβI and S100A4 expression, ferroptosis in HCC cells was significantly inhibited, and Fe^2+^ in cells was reduced; silencing the expression of the two promoted the ferroptosis process of HCC and activated the release of Fe^2+^, confirming that TGFβI and S100A4 have a significant regulatory effect on ferroptosis in HCC. These results are also consistent with those of Wang B [28] and Yang M [29], which can lay a more reliable foundation for the future diagnosis and treatment of TGFβI and S100A4 in HCC. However, the complete mechanism by which TGFβI and S100A4 regulate ferroptosis in HCC is not yet fully understood, and more comprehensive experiments are still needed for verification.

Besides, as the number of included cases is relatively small, we need to expand the sample size of the study to obtain more accurate diagnostic efficacy and cut-off values of TGFβI and S100A4 for HCC. In the follow-up research, the mechanism of action of TGFβI and S100A4 also needs to be verified through other HCC cell lines.

## Conclusion

TGFβI and S100A4 are abnormally upregulated in HCC, promoting the progression of HCC by activating ferroptosis of HCC cells and inhibiting mitochondrial oxidative stress damage. The combined detection of the two exhibits excellent evaluation effects on the occurrence and prognosis of HCC. It is expected to become a new diagnosis and treatment option for HCC in the future.

## Dodatak

### Funding

None.

### Ethics statement

The study involving human subjects complied with the Declaration of Helsinki and was approved by the ethical committee of the Second Hospital of Nanjing, Nanjing University of Chinese Medicine (No. 2022-LY-js004).

### Data availability statement

The original data presented in the study are included in the article. Further inquiries can be directed to the corresponding authors.

### Author contributions

YF.Z designed the studies, L.Z drafted and modified the manuscript, QH.S performed the statistical analysis and its design, and BW.S participated in the acquisition, analysis, or interpretation of data. M.W. participated in collecting data. All authors read and approved the final manuscript.

### Acknowledgements

None.

### Conflict of interest statement

All the authors declare that they have no conflict of interest in this work.
